# Post-traumatic acute Marjolin ulcer with a short latency period in a young man: a case report

**DOI:** 10.1186/s13256-025-05314-y

**Published:** 2025-09-29

**Authors:** Betty Kaitira, Mariam Uledi, Alex Mashaka, Maria Beda, Emmanuel Lugina

**Affiliations:** 1 Ocean Road Cancer Institute, Dar Es Salaam, Tanzania; 2 Muhimbili University of Health and Allied Sciences, P.O. BOX, 1370 Mwanza, Dar Es Salaam, Tanzania; 3 Bugando Medical Center, Mwanza, Tanzania

**Keywords:** Acute onset, Marjolin ulcer, Squamous-cell carcinoma, Short latent period, Wound

## Abstract

**Background:**

Marjolin ulcer was first mentioned by Celsius in the first century as a malignant transformation of burn scars, which can be caused by chronic wounds. Marjolin ulcer has been observed to have a latent period of over 10 years and occasionally even 36 years, with a small number of reports of short latent periods. Squamous-cell carcinoma (SCC) is the most common histology and is often characterized by an ulcerated wound around the injury site with raised margins. Marjolin ulcer squamous-cell carcinoma’s poor vascularity makes it difficult for it to respond to chemotherapy and radiotherapy. There is lack of data on acute Marjolin ulcer related to post-traumatic wounds in sub-Saharan Africa. We are reporting on a rare case of acute Marjolin ulcer squamous-cell carcinoma of the lower limb that was treated at Ocean Road Cancer Institute in Tanzania. The authors sought to enhance awareness of acute Marjolin ulcer in low- and middle-income countries.

**Case description:**

This is a case of a 35-year-old male patient of African origin who was working as an agriculture officer and was involved in a motor bicycle traffic accident 6 years prior at the age of 29 years and sustained facial, chest, and right lower limb injuries, which healed by secondary intention 3 months later. A total of 6 months later, he was diagnosed with invasive squamous-cell carcinoma around the lesions in his right knee. The patient was treated with surgery, chemotherapy, and radiotherapy.

**Conclusion:**

To our knowledge, this is likely to be the first reported case of acute Marjolin ulcer in East Africa and the third in Africa. This highlights the challenges in diagnosing and managing acute Marjolin ulcer in low- and middle-income countries. The short latent period of acute Marjolin ulcer in Africa necessitates genetic and immunological studies. Where possible, deep chronic wounds should be grafted, while unstable scars should be excised and grafted.

## Introduction

Aurelius Cornelius Celsius observed the first occurrence of malignant transformation in burn scars in the first century of 100 AD. About 1700 years later, in 1828, Jean Nicholas Marjolin, a French surgeon, described this type of ulcer, which bears his name, as a post-burn ulcer with warty changes that occur in the legs. At the time, he was unaware of the malignant nature of these transformations. In 1839, Dupuytren was the first to observe the possibility of new malignancies developing in chronic wounds. The term “Marjolin’s ulcer” (MU) was first used by Da Costa in 1903 to describe tumors that arise from simple leg ulcers [1]. At first, the term “MU” was meant to refer to squamous-cell carcinoma that arose from the malignant transformation of ulcers after a burn. However, as a result of an in-depth study of the malignant transformation of other cellular components in scars after burns and case reports on the malignant transformation of scars caused by non-burn wounds, the current concept of MU encompasses all malignant tumors that primarily occur in body surface ulcers [2].

The most common histological tumor type is squamous-cell carcinoma (SCC), but basal-cell carcinomas, melanomas, and sarcomas have also been described [2]. Other rare histological tumors, especially in sub-Saharan Africa (SSA), include Kaposi’s sarcoma in one patient and a high-grade epithelioid angiosarcoma [3]. MU is more aggressive than non-MU SCC, with a higher potential for early metastasis [3]. Cutaneous non-MU SCC is related to excessive sun exposure, radiation, genetic disorders (e.g., xeroderma pigmentosum), and immunosuppression [3]. MUs occur in burn scars, with the incidence of burn-scar neoplastic degeneration reported at between 0.77% and 2.0% [4]. However, many types of injuries can transform into MU, including bite wounds, traumatic penetrating injuries such as knife or gunshot inflictions, frostbite, vaccination sites, and pressure ulcers [5]. In SSA, burn scars, chronic ulcers, osteomyelitis, and ‘‘other’’ ulcers constituted 49%, 42%, 5.4%, and 3.6% of MUs, respectively, and 69% of MUs occur in lower extremities [6]. MU has been reported to result from genital ulcer disease [7].

Most reports show a male preponderance, with male-to-female ratios of 2–3:1 reported [4–7]. Male preponderance to trauma and poor management of resulting wounds may explain a high male-to-female ratio [1]. In SSA, the incidence of MU is low, perhaps due to underreporting [6]. MU is uncommon in high-income country (HIC) settings. However, it may be more common in low- and middle-income (LMIC) settings, given the role of chronic wounds arising from situations that involve suboptimal management at the time of primary injury [8].

The latent period is defined as the time from initial injury to the diagnosis of MU. Chronic or classic MUs have latent periods of 1 or more years; the latent period is inversely proportional to the patient’s age at the time of injury [6]. The latent period, until the malignant transformation of chronic MUs, takes on average 30 years [9]. Acute MUs, with a latent period of less than 1 year, have also been reported; they constitute about 4.6% of patients with MUs. These may occur as early as 6 weeks after injury and are more likely to occur in younger patients (40 ± 19 years old) [10]. The acute MU with the shortest latent period has been reported by Henderson *et al*., which occurred 4 weeks after a dog bite [5]. The average latent period for all sub-Saharan African patients is 16 years [3]. However, the latent period seems to be getting shorter in low- and middle-income countries, including those in Africa [11]. In Africa, only two cases of acute MU have been reported, and both are from Nigeria [7, 12].

Marjolin ulcers have been reported in every age group but occur predominantly in middle age, with a reported mean age of 75 ± 2.5 years in HIC [2]. The age of onset is, however, thought to be lower in LMIC, being 48 years in SSA [6]: 42 years in Nigeria [12] and 38 years in Tanzania [11].

We are reporting on an acute MU case in a 35-year-old man, who was 29 years old at the time of initial injury with a latent period of 6 months. To the best of our knowledge, acute MU has not been reported in East Africa before, and this is only the third reported case in SSA.

### Case presentation

**Fig. 1:**
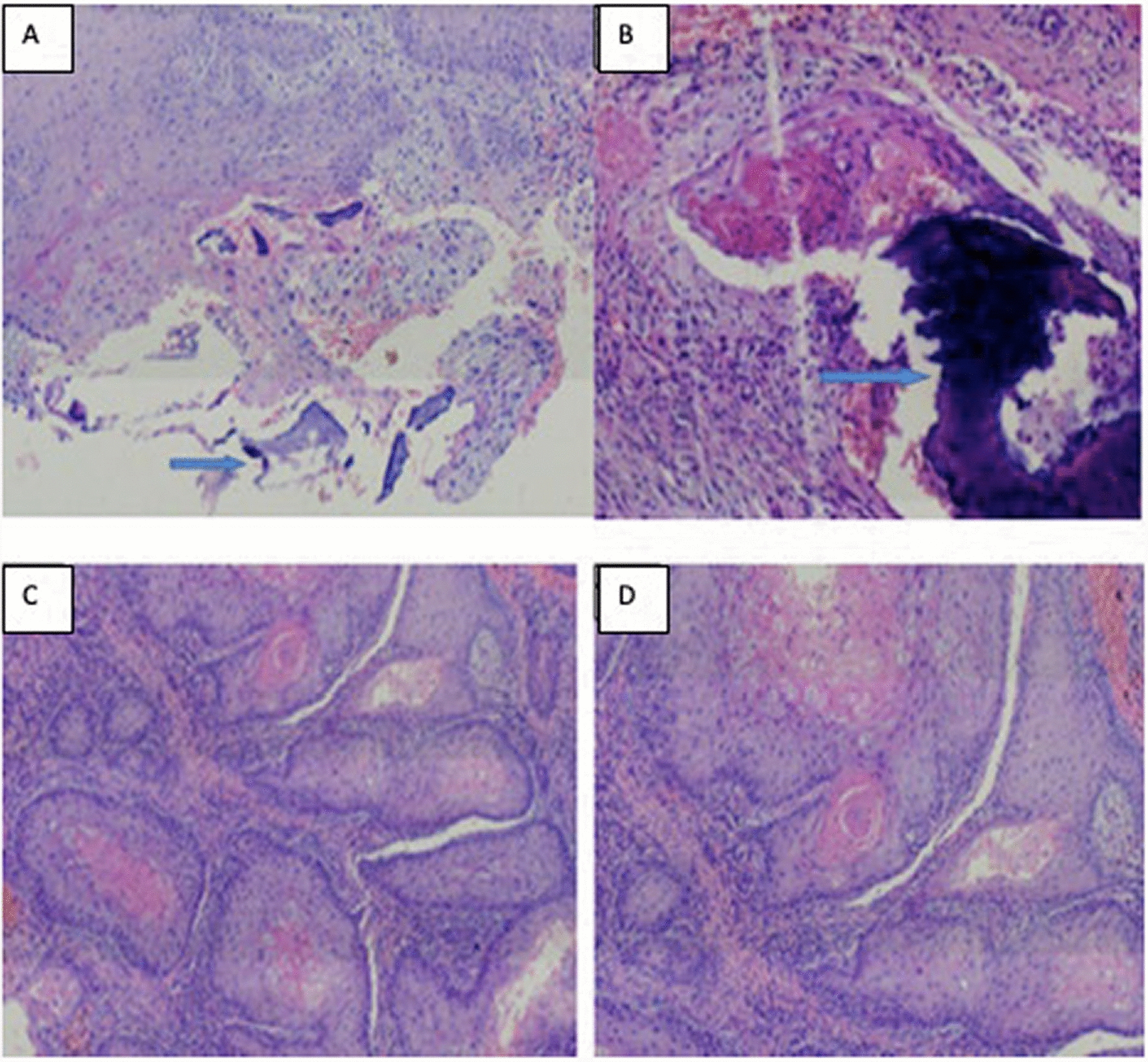
**A**–**D** Haematoxylin and eosin-stained sections. **A** and **B** Tumor nests of atypical squamous cells destructing the bone, indicated by arrows (×100 magnification). **C** and **D** A well-differentiated squamous-cell carcinoma forming keratin pearls (×100 magnification)

A 35-year-old male agriculture officer of African origin was involved in a motorcycle traffic accident in September 2018 and sustained face injuries, ribs injuries, clavicle closed fractures, and right lower limb injuries around the knee. He was treated for 3 months and healed with scars. A few months later, the scars on the right knee started to itch, and eventually ulcerated right lower limb x-ray was negative for chronic osteomyelitis. A bone and soft-tissue biopsy was taken 6 months after the accident when the patient was 29 years of age and revealed invasive squamous-cell carcinoma (SCC) (Fig. 1).

**Fig. 2:**
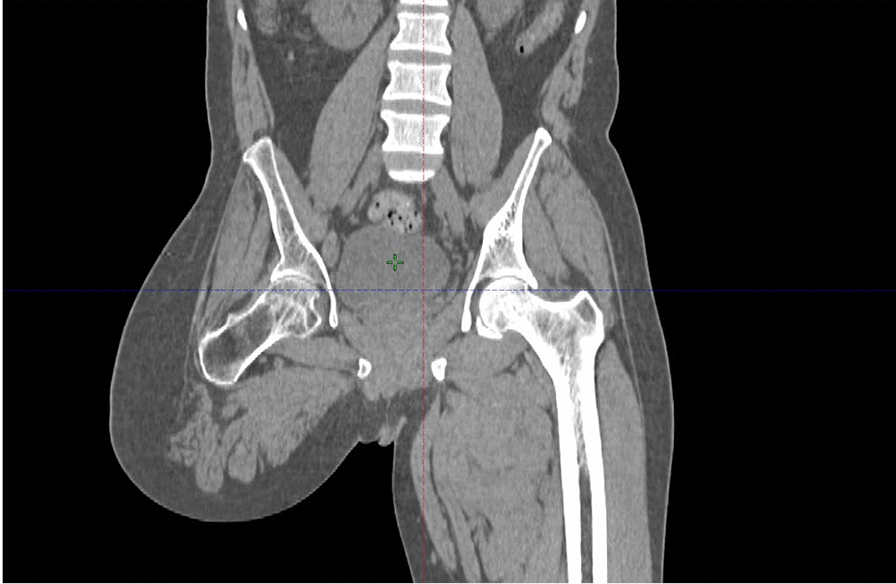
Coronal view of computed tomography scan of the pelvis showing transfemoral amputation

The decision was made to amputate his right lower leg, but he declined and opted to visit another hospital. At that hospital, he was treated with four cycles of chemotherapy (paclitaxel 350 mg and carboplatin 200 mg administered three times weekly), followed by 25 sessions of external beam radiotherapy (three-dimensional conformal radiotherapy [3DCRT] 50 Gy/25 fractions) to the lesion, with partial response. After a year, the lesion worsened, and a transfemoral amputation was performed (Fig. 2).

**Fig. 3:**
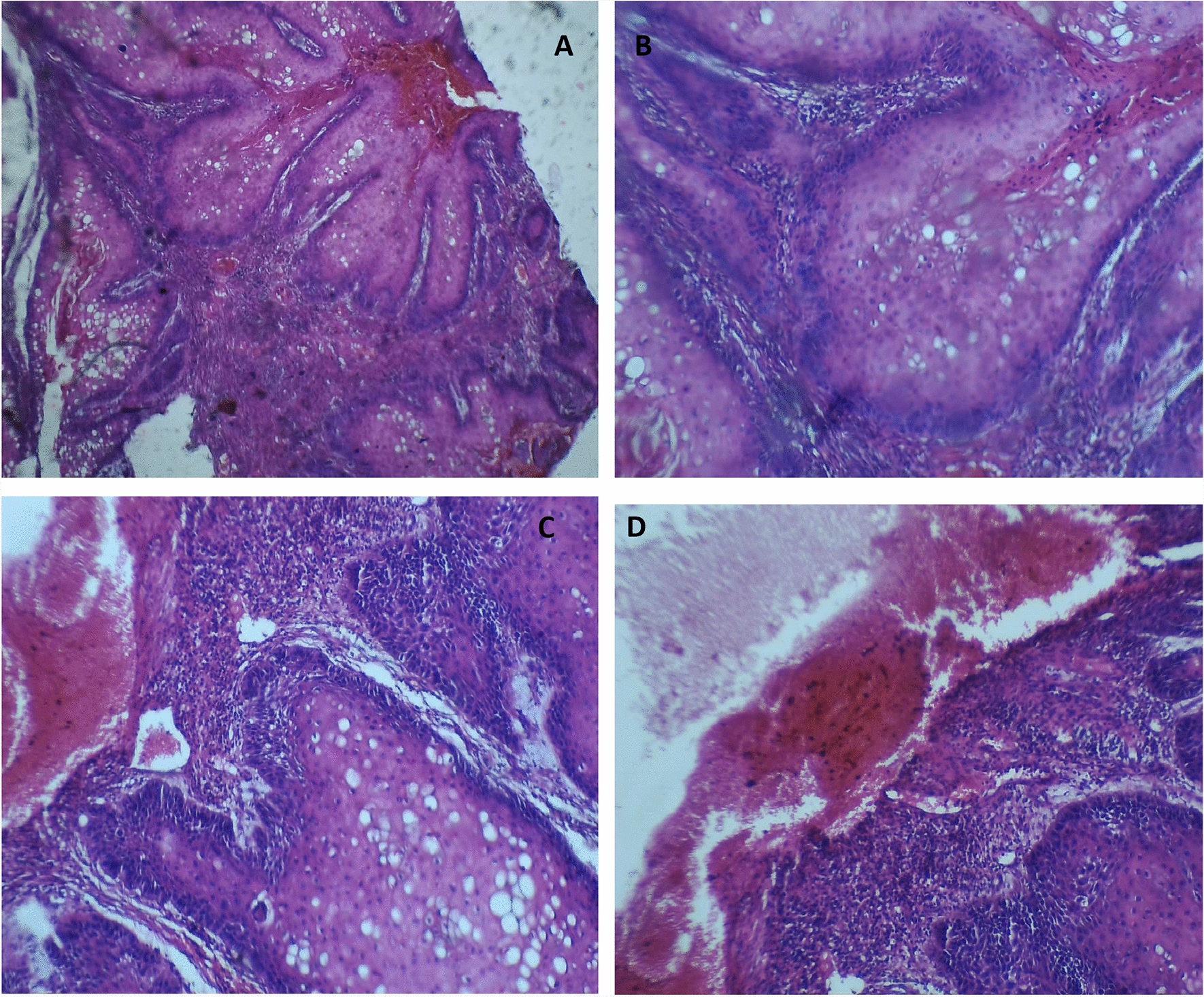
**A**–**D** Haematoxylin and eosin section showing dysplastic skin tissue with hyperplastic epithelium with areas of glycogenizations with pleomorphism of the epithelial cells (**A** and **B**). **C** Tumors nested with stromal invasion by neoplastic cells with dyskeratosis. **D** Breach of the basement membrane by neoplastic cells and scattered single neoplastic cells in the reactive stroma with area of hemorrhage and inflammatory cell infiltrate (×400 magnification)

A total of 9 months later, he developed ulcerated matted inguinal lymphadenopathy in his right groin. A biopsy from the skin around the inguinal region revealed invasive squamous-cell carcinoma (SCC) Grade I, as shown in Fig. 3.

The computed tomography (CT) scans of the chest, abdomen, and pelvis found no abnormalities. He was given four cycles of capecitabine 2000 mg orally twice a day for 2 weeks with 1 week off, but there was no response.

In December 2023, the patient decided to self-refer to our facility. He reports a positive history of exposure to chemical compounds (weeds, pesticides, and veterinary drugs) before and after the accident but denied a history of similar illness or past chronic illness. The patient has no history of exposure to smoking, alcohol use, or radiation before developing the disease. The other family members do not have any history of illness of a similar nature.

On examination, he was alert, afebrile, not pale, not dyspneic, not cyanosed, and not jaundiced, with no lower limb edema and with right limb transfemoral amputation. His blood pressure was 135/76 mm Hg, pulse rate 105 beats per minute, temperature 36.5 centigrade, respiratory rate 18 breath per minute, and oxygen saturation 97% on room air during the general examination. His functional status was an Eastern Cooperative Oncology Group (ECOG) score of 1. During the local examination, it was found that there was a large ulcer located around the right inguinal region, with a poorly defined margin and foul-smelling discharge. He had bilateral inguinal lymphadenopathies, which were fixed and matted. The patient’s Glasgow Coma Scale score was 15/15. The examination revealed that all other systems were normal, including the central nervous, chest, cardiovascular, and gastrointestinal systems.

**Fig. 4:**
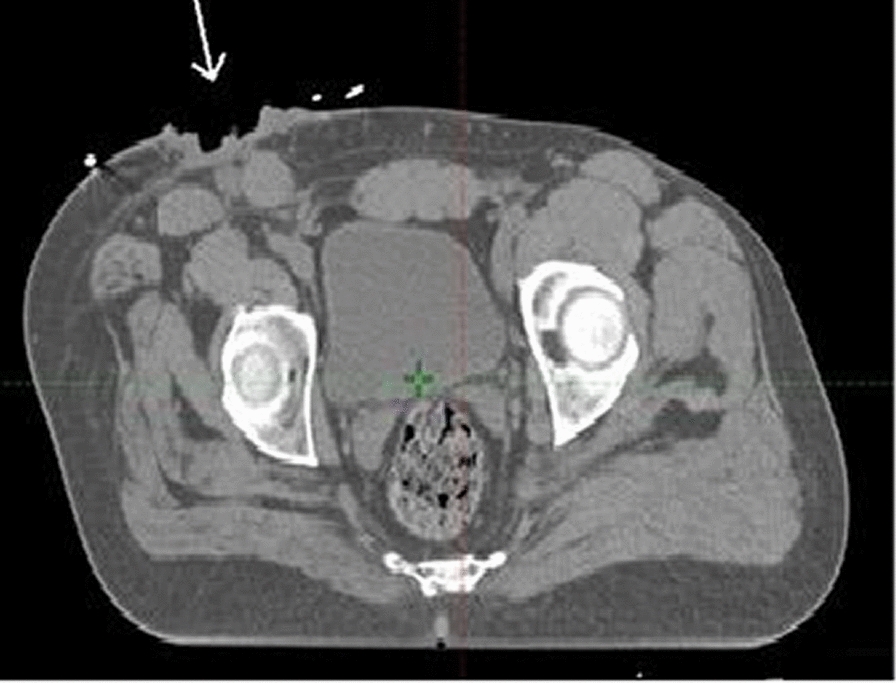
A pelvic cross-sectional computed tomography scan image showing an ulcerated right inguinal nodal mass lesions of about 83 mm × 40 mm (white arrow) infiltrating the anterior muscle complex with other multiple enlarged ipsilateral inguinal lymph nodes

Human immunodeficiency virsus (HIV)-1 serology was negative. The hemoglobin level was 16.1 g/dL, and the white blood cell (WBC) count was in the normal range. CT scan of the pelvis revealed malignant right inguinal nodal mass lesions of about 83 mm × 40 mm infiltrating the anterior muscle complex with other multiple enlarged ipsilateral inguinal lymph nodes, the largest measuring 23 mm × 20 mm. A pelvic CT scan also revealed 17 mm × 12 mm ovoid lymph nodes that were homogeneously enhancing in the left inguinal region, as shown in Fig. 4.

A CT scan of the chest and abdomen showed no significant abnormalities. The CT scan of the total spine revealed a T12 osteolytic lesion along the vertebral body highly suggestive of osseous metastasis. There were also bilateral enlarged inguinal lymphadenopathies with ulcerative soft-tissue mass along the exposed right sartorius, rectus femoris, and tensor fascia lata muscles.

**Fig. 5:**
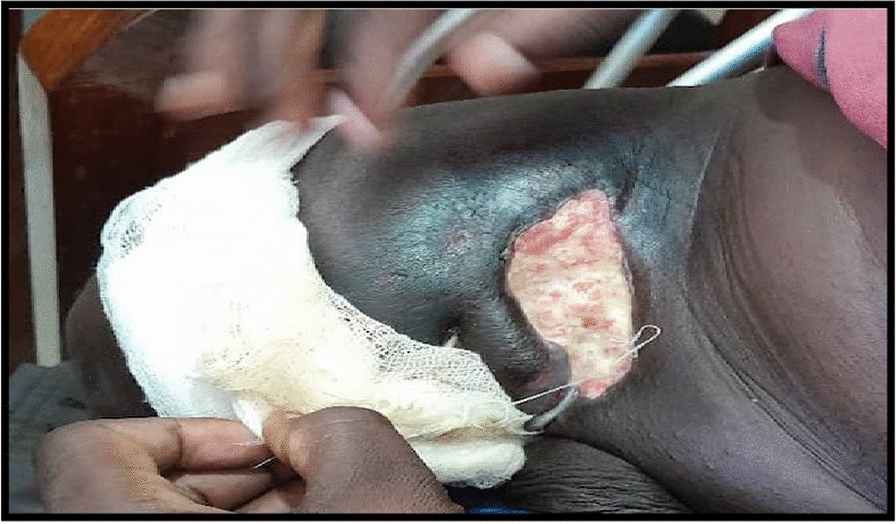
Appearance of the inguinal lesion following external beam radiotherapy

**Fig. 6:**
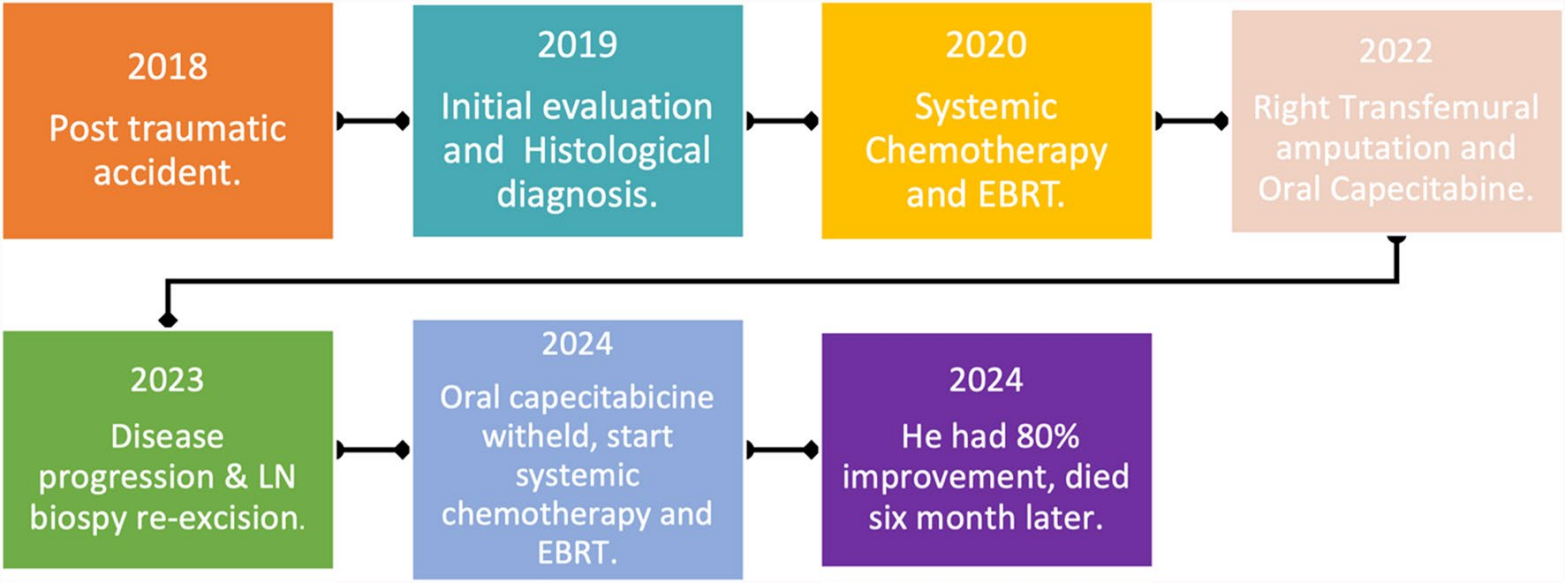
A timeline of significant events

The patient was staged according to American Joint Committee on Cancer (AJCC) Eighth Edition tumor, node, metastasis (TNM) staging as T3N3bM1. The disease progressed while he was waiting for radiotherapy. It was decided that he should undergo induction chemotherapy with carboplatin 150 mg intravenously and paclitaxel 160 mg intravenously once a week for six cycles. He was only able to receive three cycles of chemotherapy because he experienced severe hypoglycemia episodes that were managed in a nearby hospital. The patient was treated with palliative photons external beam radiotherapy (3DCRT 50 Gy/25 fractions) at our center with 6 MV for anterior–posterior field energy and 15 MV for posterior–anterior field energy with gantry angles rotated to 180°, and he obtained a remarkable response of approximately 80% (Fig. 5). However, after 6 months of follow-up visits, the patient died. A summary in Fig. 6 highlights important events.

## Discussion

A chronic leg ulcer that has hyperkeratotic granulation, changes or a raised margin, unusual pain, bleeding, or a protracted course despite appropriate treatment may be showing signs of malignant transformation [9]. Itching is another sign of malignant transformation, as observed in this case report [3]. Another possible theory for the mechanism and rapidity in which the injury may turn cancerous is due to chronic irritation or repeated trauma leading to a diminished epithelium.

There are multiple theories about the malignant transformation of scar tissue currently [1]. One possible mechanism is reduced vascular supply to the scarred area. Local fibrosis’ decreased blood flow hinders the removal of carcinogenic material and hinders the immune system’s ability to detect and destroy abnormal cells [5]. Another possible theory for the mechanism by and rapidity at which the injury may turn cancerous is chronic irritation or repeated trauma leading to a diminished epithelium [13]. Furthermore, the wound area near the joint experiences frequent flexion and extension, which results in repeated stretching of the skin, leading to chronic irritation and a cycle of damage and repair [5]. Our patient’s scar was located around the knee.

The environmental and genetic interaction theory seeks to explain the evolution of acute MU. It suggests that genetic differences increase the susceptibility to environmental insults, leading to a short latent period [10]. Our patient was an agriculture officer who had been exposed to chemical compounds (weeds, pesticides, and veterinary drugs) for a long time, and perhaps he had some genetic traits that predisposed him to chemical insults. Indeed, the genetic theory was proposed after *p53* and *Fas* gene mutations were found in patients with Marjolin’s ulcers [14]. Studies have also indicated that patients with MU have a reduced T-cell count, suggesting that immunosuppression is a contributory factor [1].

Our patient was diagnosed at the age of 29 years, and the latent period was only 6 months. Studies have shown that younger patients (40 ± 19 years old) are more likely to have a latent period of less than 1 year, while elderly patients (50 ± 18 years old) tend to have a latency period of more than 1 year [10]. The challenge in cases with short latent periods is to prove the absence of cancer in the tissues prior to the development of the observed malignant transformation. Indeed, some have argued that acute MU may actually have arisen from skin that previously had a premalignant change [1]. There is a possibility that our patient’s skin had co-existing premalignant change as a result of chronic exposure to chemicals.

MU is more likely to metastasize than non-MU SCC [16, 17]. Our patient developed bone metastasis, which is quite rare in non-MU SCC.

The only curative treatment for MU is surgery. The procedure necessitates a wide local excision and skin grafting or amputation if the lesion is inoperable [18]. Wide surgical margins of up to 2–4 cm have been suggested by various authorities [19–21]. Our patient needed wide local excision and skin grafting when the scar started to itch. There was also a delay in amputating the leg when the lesion was deemed inoperable by wide local excision.

The role of systemic therapy for MU is not well defined in the literature, and its use is quite scattered. The benefit of concurrent systemic therapy with radiotherapy is unclear, as is the potential use of systemic therapy (and/or radiotherapy) as a neoadjuvant treatment to facilitate surgical removal [15]. The relatively poor vascularity of these cancers may also explain their inadequate response to systemic chemotherapy [5]. Although the prognosis is excellent for low-risk tumors, high-risk lesions are associated with increased mortality and require lifelong monitoring [11, 21–26].

The main reasons for this case report’s late presentation were a lack of awareness and a low index of suspicion. Greater attention should be given to patients with wounds that heal by secondary intention, wounds that do not heal appropriately, and fragile burn scars that ulcerate easily. It is crucial for these patients to seek medical attention and avoid delaying definitive treatment quickly, as we have witnessed in our case.

Radiotherapy and chemotherapy have not been shown to be effective in treating Marjolin ulcers. However, radiotherapy may be used for palliation in cases where surgery is not possible or has been refused [15]. The poor vascularity of MU may explain the inadequate response to radiotherapy and chemotherapy [1].

This case report’s strength lies in its detailed elaboration of significant events. Although the authors attempted to capture all the case reports and series of acute MU from Africa in the literature, they probably missed some that were not captured through PubMed, case reports and series references, and the Internet or that were otherwise unavailable because of a language barrier.

## Conclusion

We have reported the first case of acute MU in East Africa and the third in Africa. This highlights the challenges in diagnosing and managing acute MU in low- and middle-income countries. The short latent period of acute MU in Africa necessitates genetic and immunological studies. Where possible, deep chronic wounds should be grafted, while unstable scars should be excised and grafted. It is prudent to have a high index of suspicion for acute MU among patients with inadequately managed wounds.

## Data Availability

Data is available upon reasonable request.

## Abbreviations

3DCRT: Three-dimensional conformal radiotherapy
HIC: High-income countries
LMIC: Low- and middle-income countries
MU: Marjolin’s ulcer
ORCI: Ocean Road Cancer Institute
SCC: Squamous-cell carcinoma
SSA: Sub-Saharan Africa

## References

[1] Pekarek B, Buck S, Osher L. A comprehensive review on Marjolin’s ulcers: diagnosis and treatment. J Am Coll Certif Wound Spec. 2011. 10.1016/j.jcws.2012.04.001. 10.1016/j.jcws.2012.04.001 PMC3601857 24525526

[2] Daya M, Balakrishan T. Advanced Marjolin’s ulcer of the scalp in a 13-year-old boy treated by excision and free tissue transfer: case report and review of literature. Indian J Plast Surg. 2009. 10.4103/0970-0358.53020. 19881030 10.4103/0970-0358.53020 PMC2772291

[3] Asuquo ME, Ikpeme IA, Bassey EE, Ebughe G. Squamous cell carcinoma in south-eastern equatorial rain forest in Calabar Nigeria. Eplasty. 2009;9:e53. 20011213 PMC2779782

[4] Sirsat MV, Shrikhande SS. Histochemical studies on squamous cell carcinoma of the skin arising in burn scars with special reference to histogenesis. Indian J Cancer. 1966;3(3):157. 5917023

[5] Novick M, Gard DA, Hardy SB, Spira M. Burn scar carcinoma a review and analysis of 46 cases. J Trauma Injury, Infect Crit Care. 1977. 10.1097/00005373-197710000-00010. 909123

[6] Callam MJ, Harper DR, Dale JJ, Ruckley CV. Chronic ulcer of the leg: clinical history. Br Med J. 1987;294:6584. 10.1136/bmj.294.6584.1389 PMC1246555 3109669

[7] Horton CE, Crawford HH, Love HG, Loeffler RA. The malignant potential of burn scar. Plast Reconstr Surg. 1958. 10.1097/00006534-195810000-00008. 10.1097/00006534-195810000-00008 13590989

[8] Nthumba PM. Marjolin’s ulcers in sub-Saharan Africa. World J Surg. 2010. 10.1007/s00268-010-0727-6. 20645092 10.1007/s00268-010-0727-6

[9] Trent JT, Kirsner RS. Wounds and malignancy. Adv Skin Wound Care. 2003. 10.1097/00129334-200301000-00014. 12582304 10.1097/00129334-200301000-00014

[10] Lim JL, Asgari M. Clinical features and diagnosis of cutaneous squamous cell carcinoma. UpToDate. 2015

[11] Nthumba PM. Marjolin’s ulcers: theories, prognostic factors and their peculiarities in spina bifida patients. World J Surg Oncol. 2010. 10.1186/1477-7819-8-108. 21129225 10.1186/1477-7819-8-108 PMC3014936

[12] Copcu E. Marjolin’s ulcer: a preventable complication of burns? Plast Reconstr Surg. 2009. 10.1097/PRS.0b013e3181a8082e. 19568055 10.1097/PRS.0b013e3181a8082e

[13] Sabin SR, Goldstein G, Rosenthal HG, Haynes KK. Aggressive squamotous cell carcinoma originating as a Marjolin’s ulcer. Dermatol Surg. 2004. 10.1097/00042728-200402000-00026. 14756658 10.1111/j.1524-4725.2004.30072.x

[14] Copcu E, Aktas A, Şişman N, Oztan Y. Thirty-one cases of Marjolin’s ulcer. Clin Exp Dermatol. 2003. 10.1046/j.1365-2230.2003.01210.x. 12653697 10.1046/j.1365-2230.2003.01210.x

[15] Abdi MA, Yan M, Hanna TP. Systematic review of modern case series of squamous cell cancer arising in a chronic ulcer (Marjolin’s ulcer) of the skin. JCO Glob Oncol. 2020. 10.1200/GO.20.00094. 32530749 10.1200/GO.20.00094 PMC7328103

[16] Dupree MT, Boyer JD, Cobb MW. Marjolin’s ulcer arising in a burn scar. Cutis. 2000;62(1):49. 9675536

[17] Duncan KO, Leffell DJ. Epithelial precancerous lesions. In: Freedberg IM, Eisen AZ, Wolff K. Fitzpatrick's dermatology in general medicine. 6 ed. New York: Mc Graw-Hill; 2003. p. 719–36.

[18] Kumar BRK, Shankar AP, Narayanan GS, Ganesh MS. Twenty-seven cases of Marjolins ulcer; an institutional experience on diagnosis, treatment and outcomes. Asia Pac J Cancer Biol. 2024;9(1):17–20.

[19] Ames FC, Hickey RC. Squamous cell carcinoma of the skin of the extremities. Int Adv Surg Oncol. 1980;3:179–99. 6926740

[20] Atiyeh BS, Hayek SN, Kodeih MG. Marjolin’s ulcer of the scalp: a reconstructive challenge. Ann Burns Fire Disasters. 2005;18(4):197–201. 21991007 PMC3188002

[21] Rowe DE, Carroll RJ, Day CL. Prognostic factors for local recurrence, metastasis, and survival rates in squamous cell carcinoma of the skin, ear, and lip: implications for treatment modality selection. J Am Acad Dermatol. 1992. 10.1016/0190-9622(92)70144-5. 1607418 10.1016/0190-9622(92)70144-5

[22] Kwa RE, Campana K, Moy RL. Biology of cutaneous squamous cell carcinoma. J Am Acad Dermatol. 1992. 10.1016/0190-9622(92)70001-V. 1732313 10.1016/0190-9622(92)70001-v

[23] Kraus DH, Carew JF, Horrison LB. Regional lymph node metastasis from cutaneous squamous cell carcinoma. Arch Otolaryngol Head Neck Surg. 1998. 10.1001/archotol.124.5.582. 9604987 10.1001/archotol.124.5.582

[24] Michalopoulos N, Sapalidis K, Laskou S, Triantafyllou E, Raptou G, Kesisoglou I. Squamous cell carcinoma arising from chronic sacrococcygeal pilonidal disease: a case report. World J Surg Oncol. 2017. 10.1186/s12957-017-1129-0. 28302173 10.1186/s12957-017-1129-0 PMC5356347

[25] Kerr-Valentic MA, Samimi K, Rohlen BH, Agarwal JP, Rockwell WB. Marjolin’s ulcer: modern analysis of an ancient problem. Plast Reconstr Surg. 2009. 10.1097/PRS.0b013e3181904d86. 19116552 10.1097/PRS.0b013e3181904d86

[26] Fairbairn NG, Hamilton SA. Management of Marjolin’s ulcer in a chronic pressure sore secondary to paraplegia: a radical surgical solution. Int Wound J. 2011. 10.1111/j.1742-481X.2011.00831.x. 21827630 10.1111/j.1742-481X.2011.00831.x PMC7950692

